# Evidence for Rhythmicity Pacemaker in the Calcification Process of Scleractinian Coral

**DOI:** 10.1038/srep20191

**Published:** 2016-02-05

**Authors:** Eldad Gutner-Hoch, Kenneth Schneider, Jaroslaw Stolarski, Isabelle Domart-Coulon, Ruth Yam, Anders Meibom, Aldo Shemesh, Oren Levy

**Affiliations:** 1 The Mina & Everard Goodman Faculty of Life Sciences, Bar-Ilan University, 52900 Ramat-Gan, Israel; 2 Institute of Paleobiology, Polish Academy of Sciences, Twarda 51/55, PL-00-818 Warsaw, Poland; 3 MCAM UMR7245, Sorbonne Universités, Muséum National d’Histoire Naturelle, (CP54) 57 rue Cuvier, 75005 Paris, France; 4 Department of Earth and Planetary Sciences, Weizmann Institute of Science, P.O.Box 26, 76100 Rehovot, Israel; 5 Laboratory for Biological Geochemistry, School of Architecture, Civil and Environmental Engineering (ENAC), Ecole Polytechnique Fédérale de Lausanne (EPFL), CH-1015 Lausanne, Switzerland; 6 Center for Advanced Surface Analysis, Institute of Earth Sciences, University of Lausanne, Lausanne, Switzerland

## Abstract

Reef-building scleractinian (stony) corals are among the most efficient bio-mineralizing organisms in nature. The calcification rate of scleractinian corals oscillates under ambient light conditions, with a cyclic, diurnal pattern. A fundamental question is whether this cyclic pattern is controlled by exogenous signals or by an endogenous ‘biological-clock’ mechanism, or both. To address this problem, we have studied calcification patterns of the Red Sea scleractinian coral *Acropora eurystoma* with frequent measurements of total alkalinity (A_T_) under different light conditions. Additionally, skeletal extension and ultra-structure of newly deposited calcium carbonate were elucidated with ^86^Sr isotope labeling analysis, combined with NanoSIMS ion microprobe and scanning electron microscope imaging. Our results show that the calcification process persists with its cyclic pattern under constant light conditions while dissolution takes place within one day of constant dark conditions, indicating that an intrinsic, light-entrained mechanism may be involved in controlling the calcification process in photosymbiotic corals.

In the marine environment, diel periodicity is mainly governed by light and dark cycles and by tidal cues^1^ that synchronize a multitude of biological processes in aquatic organisms. Basal metazoan organisms, such as scleractinian corals, offer insights into the origins of the circadian machinery that regulates temporal patterns throughout the animal kingdom. In addition, scleractinian corals are among the most efficient bio-mineralizing organisms in nature, forming vast coral reefs in the shallow waters of the tropical and sub-tropical oceans^2^. Reef-building corals are furthermore interesting for their symbiosis with dinoflagellates of the genus *Symbiodinium sp.* (commonly named zooxanthellae), that drive major diurnal changes in their tissue, via their influence on oxygen tension, pH, nutrient fluxes, and other variables^3^,^4^,^5^,^6^,^7^. Although there are strong indications of rhythmic behavior in corals^8^,^9^, very little is known about the circadian clock mechanisms that control the biology of these symbiotic organisms. Recently, molecular and physiological studies have demonstrated that circadian mechanisms are involved in the control of the host metabolism^10^,^11^ and the symbionts photosynthesis^12^,^13^,^14^.

Studies of abiotic impacts on the coral calcification process have focused mainly on seawater temperature^15^,^16^,^17^, carbonate saturation state^18^,^19^,^20^,^21^, and light^22^,^23^,^24^,^25^. However, the tuning of the process is not clear. The calcification process is generally linked to the day-night cycle, with higher calcification rates observed during the daytime (so-called Light-Enhanced Calcification^26^,^27^,^28^), although this is still a matter of some debate^29^. A few studies have suggested that the daily rate of calcification is regulated by an intrinsic rhythm^30^,^31^, without specifying the possible mechanism.

The circadian clock is an endogenous chronometer found in most eukaryotes and in photosynthetic bacteria^24^. The clock drives rhythms that regulate the physiology, biochemistry, and metabolism of the organism^25^, and is coupled to the environment via synchronizing cues, or zeitgebers (time-givers), such as light and temperature cycles. These environmental cycles allow organisms to maintain robust rhythms with a 24 hours periodicity over a broad range of physiological, temperature, and light regimes^26^. Circadian rhythms are generally considered to be free running, maintaining a periodicity of ≈24 hours for some time under constant stimuli or in the absence of external diel cues; for example in constant light, constant darkness, or constant temperature. In order to understand if the calcification process in corals is controlled by an endogenous pacemaker, synchronized primarily with light intensity, a series of 48 h experiments were conducted with the coral *Acropora eurystoma* (Fig. 1) under four light regimes: 1) constant light (LL), two 24 h periodic light regimes consisting of 2) the ambient natural cycle and 3) 10 h/14 h light /dark (LD), and 4) constant darkness (DD). During these different light treatments the calcification rates of the corals were measured. In addition, repeated *in-situ*^86^Sr-isotope labeling was performed under the various experimental conditions to assess if mentioned light treatments affect the skeletal extension and skeletal ultrastructures.

## Results

### Relative calcification cycles

The measurements of relative variations in total alkalinity (A_T_) during successive two-hour incubation periods show that the tropical symbiotic coral *A. eurystoma* maintained a cyclic and rhythmic pattern of calcification under all light regimes tested (Ambient, LD and LL), with dissolution during night-time and throughout DD (Fig. 2). The rhythmic pattern of calcification cycle under LD treatment (Fig. 2b) was similar to the calcification pattern under ambient light, showing significant maximum during the midday hours in both light periods (repeated measures ANOVA followed by Bonferroni test, *F*(11, 33) = 521.878, *p* < 0.005 and *F*(11, 33) = 163.854, *p* < 0.0005 for ambient and LD treatments, respectively). This result is in spite of the fact that the artificial light had a constant irradiance and during LD runs light was switched on and off instantaneously compared to gradual irradiance change in nature.

The calcification cycle under LL conditions (Fig. 2c) showed a rhythmic calcification pattern with a free run period close to a 24 hours cycle. During the subjective night-time, relative calcification values were low and approached dissolution. The significant calcification maxima occurred at 12:00 (midday) on the first day under LL while on the subsequent day, maximum calcification was observed between 12:00 to 20:00 (repeated measures ANOVA followed by Bonferroni test, *F*(11, 44) = 24.24, *p* < 0.005). Calcification of coral fragments kept under the LL treatment increased at the beginning of the second day in the subjective second dawn. In the second subjective night, relative calcification values did not decrease, as they did for ambient and LD (second time-point 20:00), but dropped later in the next measurement time at 0:00 (second subjective midnight). In the third morning, the calcification of the coral fragments kept in the LL treatment rose significantly more than those kept in ambient and LD. Although under LL treatment in the second 24 hours a prolonged calcification pattern was observed, no difference was identified in the overall calcification pattern in the first 24 hours, between the three light treatments (ambient, LD and LL). During DD, only net dissolution was observed and the relative dissolution values were significantly different only between the peak of dissolution on the second day (20:00) and minimum dissolution at midnight (00:00) and midday of the second day (repeated measures ANOVA followed Bonferroni test, *F*(11, 22) = 7.675, *p* < 0.005).

### Dynamics of skeletal extension visualized by ^86^Sr label incorporation

Successive growth fronts labeled with ^86^Sr were visualized with NanoSIMS ion microprobe imaging in sections of skeleton of coral nubbins (Fig. 3). Corals grown under the ambient LD photoperiod (Fig. 3a–c) or under the LD and LL light treatments (Fig. 3d–f) typically exhibited all five ^86^Sr bands. The labeled growth fronts passed mostly through the Thickening Deposits (TD), which represent the bulk of the skeleton, and sometimes through the Rapid Accretion Deposits (RAD), which are formed on the growing skeletal tips. Labeled RAD were especially visible in the constant light treatment (Fig. 3f), forming a continuous growth front with adjacent TD. These results indicate that both of these ultra-structural components of the skeleton are forming during the 12 hours daytime labeling pulses, both under the LD photoperiod and under the constant light (LL) conditions.

Under the constant light treatment, the ^86^Sr-labeled RADs formed during the 4^th^ labeling pulse were thicker compared to the adjacent Thickening Deposits (TD) formed in the same time span (Fig. 3d–f). These observations may indicate differences in skeletal accretion rates, with a higher rate of skeletal extension in the RADs compared to the TDs, which should be confirmed via absolute Sr/Ca analysis (similar confirming results in other species *Pocillopora damicornis*^32^ and *Porites lobata*^29^). Average 12 h daytime extension rates of the Thickening Deposits, measured as the thickness of bands with incorporated ^86^Sr label, were at 0.58 μm and 0.55 μm for LD and LL, with standard deviation of 0.20 μm and 0.40 μm for LD and LL, respectively. In the constant dark treatment (DD), a maximum of three ^86^Sr labeled bands were detected in the skeleton of *A. eurystoma* nubbins (Fig. 3g–i), despite the fact that more than 10 areas in apical corallites were mapped with the NanoSIMS. This result indicates that skeletal extension continued during the first 12 hours daytime ^86^Sr pulse in the dark (labeling event L3), in at least a few skeletal areas corresponding to Thickening Deposits, and then slowed down and stopped sometime during the next 2 days under the DD treatment.

The scale-like structures (shingles) that form thickening deposits (typical of *Acropora* species) in specimens cultured under DD differ from those grown in the controlled LD and LL settings. The shingles from skeletons formed in DD are less regular and their surfaces are smoother compared to shingles from the LD and LL specimens, which are regular and their edges are more distinct (Fig. 4). The NanoSIMS maps of elongated skeletal tips (Fig. 3d–f) show distinct spatial distribution-pattern differences in ^86^Sr label incorporation between RADs and Thickening Deposits (shingles): the labeled regions close to RAD typically form continuous layers whereas in TD region, successive, crescent-like labeled zones are formed along bundles of fibers that project on the skeleton surface as shingles.

## Discussion

The majority of studies dealing with the calcification mechanism of scleractinian coral have been focused on short term observations with little attention to the calcification process diel variations^33^. In this study we investigate the calcification of the Red Sea scleractinian coral *A. eurystoma* during different light regimes treatments, with continuous observations longer than 24 hours. Throughout the constant light treatment (LL), *A. eurystoma* coral’s fragments exhibited a cyclic pattern over the first 24 hours of the experiment and then exhibited a second cycle during the second day of the experiment, where the high day calcification is prolonged (Fig. 2c). Moreover, during the constant irradiation profile of the LD illumination treatment the coral’s calcification process displayed a cyclic pattern (Fig. 2b). These calcification patterns which were exhibited under LL and LD treatments, demonstrate an interesting cyclic dynamics which is associated with the “Light-enhanced calcification” phenomenon. During exposure to light it is possible that products of photosynthesis by the zooxanthellae may affect the host calcification and act as a major energy fuel for the process^4^,^34^. Therefore, it may be the cyclic metabolic activity of the coral symbionts and their photosynthesis pacemaker^12^,^13^,^14^ that drive the cyclic pattern of the coral calcification.

Evidences from precipitation of CaCO_3_ that were recorded under DD conditions with ^86^Sr labeling of the Thickening Deposit microstructural components (Fig. 3g–i) show labeling formation that took place only during the first 12 hours of darkness. During DD treatment in the ^86^Sr labeling, the skeleton extension most likely persisted only during the first 12 hours during DD treatment (labeling event L3, which followed the last regular nighttime – Fig. 3g–i). This fact suggests that skeletal extension persisted until energy reserves that originate from photosynthate translocation by dinoflagellates were depleted. Indeed, previous work reports depletion of photosynthetic carbon reserves within 18–20 hours of constant darkness^7^. This finding indicates that the calcification process keeps going during the first subjective daytime without external light and without new photosynthate production by the algal symbionts. The SEM images of coral skeleton textures under DD (Fig. 4) clearly support the irregular extension seen with the ^86^Sr labeling. These textures imply for a possible overall CaCO_3_ dissolution over precipitation. The disagreement between the skeletal extension visualized with ^86^Sr labeling during the first 12 hours of prolonged darkness (Fig. 3g–i) and the dissolution pattern indicated from the relative alkalinity depletion (Fig. 2d) could be explained by differences in skeletal density and the fact that skeletal microstructures were not labeled in all growth directions. Calcification is the product of skeletal extension and density increase^35^, and these precipitation processes may be temporally uncoupled. It is also possible that in the absence of light, the dissolution process masks the low calcification diel cycle (Fig. 2d).

The calcification cyclic patterns during light treatments (Fig. 2a–d) presented here suggest for a link to the algae’s circadian clock, which may influence the host calcification apparatus. However, several evidences indicate for a lower degree of the symbionts involvement in the host calcification process: (a) The growing tips of branching corals show high rates of skeletal extensions, but they are free of zooxanthellae^27^,^36^,^37^; (b) Calcification rates of bleached corals were higher when exposed to light than corals with symbionts that exposed to oxygen and glycerol under dark conditions^4^; (c) Light has shown to affect the activity of transmembrane ion pumps and channels associated with the calcification mechanism of the symbiotic giant clam *Tridacna squamosa*^38^. It seems from those studies that light is important for the calcification mechanism not only as an autotrophic source of energy but as an entrainment (synchronizing) cue. Indeed, up-regulation of related genes associated with calcification has been found in skeletal organic matrix genes^39^ and also in genes of enzymes which active in the growing tips of branching corals^14^,^40^. Hence, the regulation of genes that are associated with the calcification mechanism is likely coordinated by light, and is reflected in diurnal-nocturnal genetic transcription variation. A potential explanation for the light entrainment of the calcification mechanism may be related to an internal pacemaker/circadian clock mechanism. Recent evidences have uncovered the presence of circadian clock molecular components in symbiontic corals and in other members of the phylum Cnidaria, which are counterparts of many other animal clock components related to light^41^,^42^,^43^.

The observations of skeletal formation dynamics based on ^86^Sr pulse labelling, showed overall similar pattern of skeleton formation during LD and LL light treatments. Both ultra-structural skeletal elements, the Rapid Accretion Deposits (RADs) and the Thickening Deposits (TD)^44^ were labelled with ^86^Sr during the subjective 12 h daytime pulses. RAD are characterized as regions of skeletal rapid accretion and are deposits of calcium carbonate crystals embedded in an organic material, while TD are more dense skeletal structures (include less organic material) with a fibrous appearance deposited outside adjacent RAD. Continuity of the ^86^Sr labeled growth front between RAD and TD microstructures (Fig. 3) indicate that these regions were forming simultaneously during daytime, in agreement with previous work, studying the extension of microstructures in *Porites lobata* skeleton, using ^86^Sr pulse-labeling with NanoSIMS analysis^29^. However, other authors have reported coral skeletal formation along a day-night cycle, based on temporal differences in microstructural density^45^ or organic matrix incorporation^46^.

Hence, this paper suggests that light is an entrainment (synchronizing) cue and most likely a gated signal to initiate the calcification process and guarantees that the CaCO_3_ precipitation will perform with a cyclic pattern. Due to the obligated complex association between the host and the symbiotic photosynthesis process, isolating the calcification process and revealing its composing parts need further studies. Furthermore, the interplay between the *Symbiodinium* core mechanism clock, which governs its photosynthesis and the host circadian endogenous pacemaker, is still unclear.

Nevertheless, this study demonstrates that the overall coral calcification rhythmicity is consistent with an independent cyclic pattern. The cyclic pattern of the coral calcification during the different constant light treatments, which emerged from results of relative calcification (Total Alkalinity measurements) and the skeletal extension with ^86^Sr pulse-labeling, most likely indicate the presence of an independent pacemaker (of the host, algae or even a synergistically cue from both animals) that controls the coral calcification process.

Symbiotic corals have a unique challenge in terms of their need to respond not only to external environmental factors but also to metabolite fluxes and intracellular stresses imposed by their symbionts. Therefore, reef-building corals have adapted to life with symbionts by coupling the expression of the calcification process to the circadian clock to effectively pre-empt the photosynthesis cycle^47^,^48^,^49^. Given that corals have maintained a stable partnership with their symbionts, subtle physiological and molecular adaptations are to be expected^11^. The *Acropora* calcification rhythmic oscillations with the dependency of light may illustrate the evolutionary flexibility of circadian regulatory systems between the coral and its *Symbiodinium* internal pacemaker and provide a paradigm for understanding other animal/algal symbioses. Comparison with calcifying symbiotic mollusks, some of which also host *Symbiodinium*, will be of particular interest.

## Methods

### Coral collection

**Figure 1:**
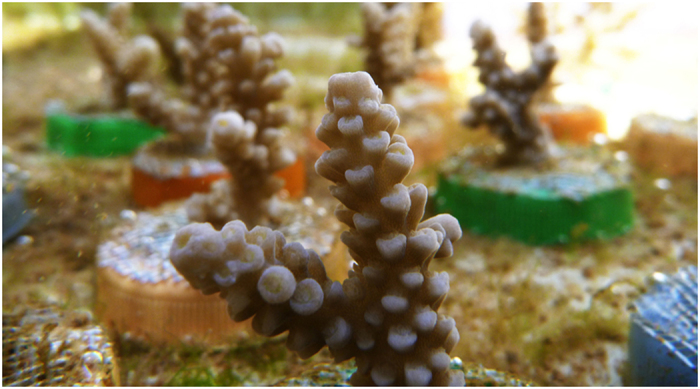
Apical branch fragments of *Acropora eurystoma*.

Apical branch fragments of scleractinian coral *Acropora eurystoma* colonies (Fig. 1) approximately 5 cm long were collected by SCUBA diving from 5–6 meters depth at the coral reef near the Inter-University Institute (IUI) in Eilat, Gulf of Aqaba, Red Sea (CITES #38406). The fragments were transferred to an outdoor, shaded running seawater table at the IUI for a month of acclimation before the experiment started.

### Relative calcification cycle measurement by total alkalinity method and data analysis

Coral calcification diel cycles were recorded under different light regimes, ambient natural light cycle, 10/14 hours of light/dark (LD), constant light (LL), and constant darkness (DD). Relative calcification values are presented as calcification measurement value relative to the highest value measured during the 48 hours experimental period for each coral fragment, according to equation 1. Calcification was determined from the difference in total alkalinity (A_T_) measured between the beginning and the end of a two-hour incubation period according to equation 2.


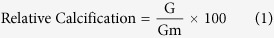



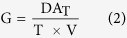


G is calcification rate and Gm is the maximum calcification rate during the 48 hours of incubations, T is the duration of the incubation (*i.e.* 2 hours) and V is the water volume in the incubation vessel. Maximum calcification (Gm) values that were used for eq. 1 were measurement time 12:00 at the first 24 hours experiment for ambient and LL treatments, measurement time 12:00 at second 24 hours experiment for LD treatment, and measurement time 20:00 at the second 24 hours experiment for DD treatment.

**Figure 2:**
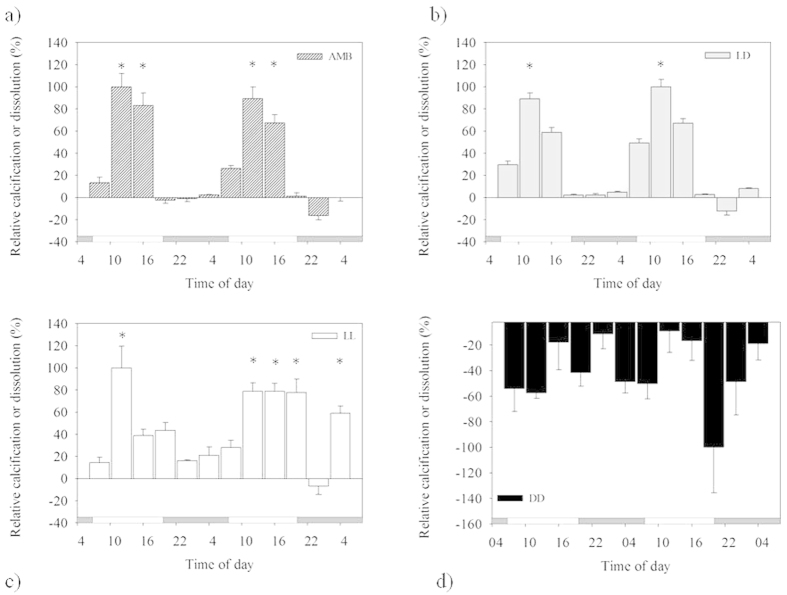
Relative calcification cycle of *Acropora eurystoma* under different light treatments over 48 hours, negative values represent dissolution. (**a**) Ambient natural light (AMB). (**b**) Light/dark (LD). (**c**) Constant light (LL). (**d**) Constant darkness (DD). Asterisks denote significant differences from other time-point measurements within the same treatment, repeated measures ANOVA, with Bonferroni test (*P* < 0.05). Error bars represent standard error.

Each incubation period was followed by a two-hour recovery period in running seawater. In this way, the relative calcification cycle was measured during a 48 hours period, with 4 fragments (quadruplicate) included in every treatment. The relative calcification cycle result during light treatments presented in Fig. 2 is the average of the 4 fragments relative calcification.

A_T_ precision is 4.6 ± 0.3 μmol Kg^−1^. Light intensity was measured using a quantum flat sensor (LI-COR, Lincoln, NE, USA). The LD and LL irradiance level was 180 ± 20 μmol quanta m^−2^ s^−1^. Seawater temperature for all treatments was 22 ± 0.5 °C. Each individual coral fragment was incubated in closed 100 ml glass jars. The incubated seawater was sampled and stored in gas tight bottles and stored in a refrigerator (4 °C) until titrated for A_T_ using Gran plot^50^ on a Titrino plus 848 automated titrator (Metrohm AG).

### Skeletal extension rate visualisation by ^86^Sr pulse-labeling and NanoSIMS imaging

**Figure 3:**
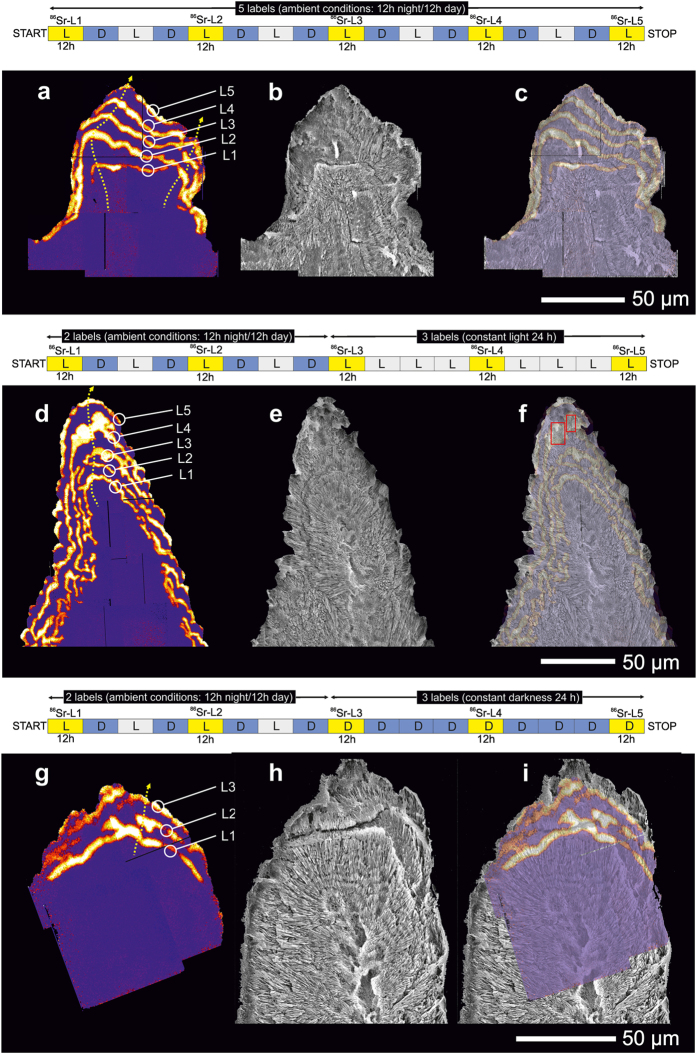
Dynamics of daytime coral skeletal extension of *Acropora eurystoma* recorded under different light treatments: ambient photoperiod (**a**–**c**), 12hLight/12hDark ("L" stands for light, 7:00 am to 7:00 pm; "D" stands for darkness, 7:00 pm to 7:00 am), constant light (**d**–**f**) (L/L) and constant darkness (**g**–**i**) (D/D). Horizontal plot above each set of images shows ^86^Sr labeling pulses (yellow boxes: 7:00 am to 7:00 pm), 36 h intervals of growth in unlabeled seawater with normal isotopic abundance, and duration of 12Light/12hDark photoperiod, constant light and constant darkness conditions. During the ambient normal photoperiod (**a–c**) and constant light (**d–f**) regimes, all 5 labels (L1–L5) are visible. During constant darkness treatments (3 last labelling events) only 3 labeling events are detectable. There is clear distinction between continuous labeling in fast skeleton extension region, which consists mostly of rapid accretion deposits and disrupted, "crescent" labeling in thickening deposits (shingles) region. NanoSIMS ^86^Sr/^44^Ca isotope mosaic maps. (**a,d,g**), SEM images of polished and etched samples. (**b,e,h**) and NanoSIMS and SEM images overlaid (**c,f,i**). Yellow dashed arrows (**a,d,g**) mark fast growing regions where Rapid Accretion Deposits (RAD’s) are mostly located. Red rectangles (**f**) mark regions where RAD are particularly clearly labeled.

**Figure 4:**
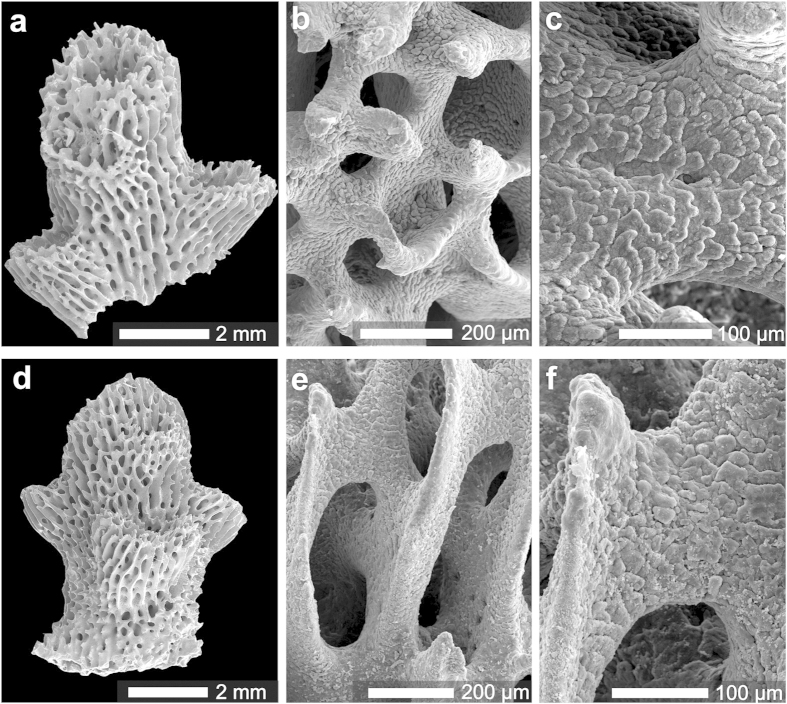
Compared skeleton surface textures of thickening deposits (*i.e.* shingles) in *Acropora eurystoma* corals cultured for 4.5 days (108 h) in constant light or constant dark conditions. (**a**–**c**) Specimen growing in constant darkness (D) during last 3 ^86^Sr labeling pulses. (**d–f**) Specimen growing in constant light (L) during last 3 ^86^Sr labeling pulses (see Figs 1 and 4). Although shingles (scale-like thickening deposits) are recognizable in both samples, shingles in specimen growing in constant light (**d–f**) are more regular and have surface with clearly visible fibers. In contrast, shingles in specimen growing in constant darkness (**a–c**) are irregular and fibers at their surface are smooth.

*A. eurystoma* coral nubbins were labeled for 12 hours in individual glass beakers containing 500 ml of seawater enriched with 10 mg/L dissolved ^86^SrCO_3_, which was prepared following the procedures in previous study^32^. A stream of air was gently bubbled in each beaker with a Pasteur pipet to mix the labeled seawater around the nubbin and to equalize oxygen and CO_2_ levels. Five successive 12 hours labeling pulses took place under three light regimes to examine the dynamics of skeletal deposition: LD, LL and DD (see also Fig. 3). Two replicate nubbins were labeled for each of the three light treatments. At the end of the fifth labeling pulse, nubbins were snap-frozen at −80 °C to stop metabolic processes. For skeletal analysis, coral tissue was removed from each nubbin using a jet of filtered seawater (Waterpik method) and the skeletons were immersed in NaClO (5%) for 20 minutes to remove residual organic matter. The skeletons were embedded in Körapox resin and sections of the apical corallites parallel to the vertical growing axis were polished. The ^86^Sr/^44^Ca distribution was mapped with a NanoSIMS ion microprobe, following established procedures^32^.

For orientation of the ^86^Sr labeling with the skeletal microstructure, Scanning Electron Microscopy (SEM) images of mildly etched skeleton were taken using FEI Philips XL 20- instrument. The same SEM was used to visualize the skeletal surface textures of samples cultured under different light treatments. Bleached skeletal branch tips were mounted on SEM stubs, platinum-coated and observed.

### Statistical analysis

For statistical analysis, repeated measures ANOVA followed by Bonferroni post hoc test was performed to assess the differences in relative calcification values within different light regimes. All statistical analyses were conducted using SPSS 20.0 (IBM, USA), and the results were considered statistically significant at *P* < 0.05.

## Additional Information

**How to cite this article**: Gutner-Hoch, E. *et al.* Evidence for Rhythmicity Pacemaker in the Calcification Process of Scleractinian Coral. *Sci. Rep.* **6**, 20191; doi: 10.1038/srep20191 (2016).
